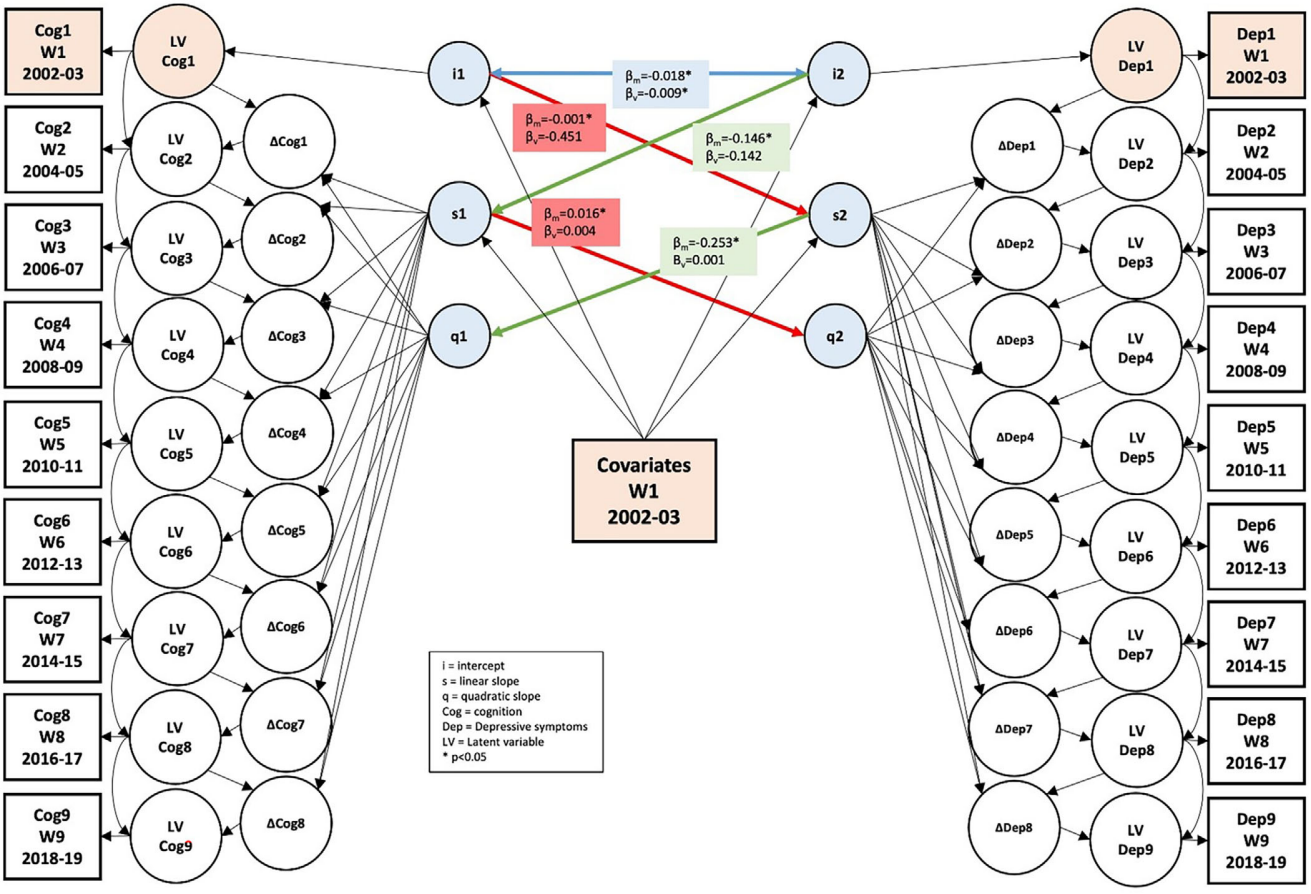# Bidirectional relation of depressive symptoms and cognitive function over 16 years follow‐up

**DOI:** 10.1002/alz.095408

**Published:** 2025-01-09

**Authors:** Jiamin Yin, Amber John, Dorina Cadar

**Affiliations:** ^1^ Department of Epidemiology and Public Health, University College London, London United Kingdom; ^2^ University College London, London, UK United Kingdom; ^3^ Centre for Dementia Studies, Brighton and Sussex Medical School, Brighton United Kingdom; ^4^ University College London, Institute of Epidemiology and Health Care, London United Kingdom

## Abstract

**Background:**

Cognitive decline and depression often co‐occur among older adults, and they share several biological mechanisms. Despite that cognitive dysfunction has been linked to depression, the directionality of this association remains unclear. This study examined whether there is a bidirectional relationship between depressive symptoms and cognitive function in middle‐aged and older English adults over a 16‐year follow‐up period.

**Method:**

The current analysis included 8,268 eligible participants from a nationally representative sample of community‐dwelling English older adults. These participants were examined longitudinally every other year from 2002 until 2018, resulting in a follow‐up period of up to 16 years. We used the bivariate dual change score models to estimate the multivariable associations between depressive symptoms and cognitive function, which are interchangeably used as exposures and outcomes. Cognitive measures include memory and verbal fluency tests, while the Center for Epidemiologic Studies Depression Scale evaluated depressive symptoms.

**Result:**

The study population (n = 8,268) had an average age of 64 years at the study baseline, and 55% of the population was female. Higher depressive symptoms were cross‐sectionally correlated with poorer memory (β intercept = −0.018, standard error (SE) = 0.004, P < 0.001) and verbal fluency (β intercept = −0.009, SE = 0.004, P = 0.02) at study baseline. A steeper linear change in depressive symptoms was related to an accelerated memory change (β intercept = −0.253, SE = 0.079, P = 0.001), and a linear change in memory was associated with an acceleration in depressive symptoms over time (β intercept = 0.016, SE = 0.006, P = 0.005). This bidirectional change was not observed with verbal fluency.

**Conclusion:**

Greater depressive symptoms were associated with poorer memory at baseline and steeper memory change over time. A gradual change in depressive symptoms contributed to accelerated memory loss and vice versa, suggesting that psychological mood and memory performance are intrinsically interrelated. In summary, these findings highlight the complex interplay between depressive symptoms and memory loss, underscoring the importance of integrated assessments and treatment approaches in clinical practice. Our work suggests that early intervention in depressive symptoms could provide a timely opportunity to slow down or delay memory decline in later life.